# The Compulsory Removal of Phthisical Patients

**Published:** 1916-06-24

**Authors:** Frederic G. D'aeth

**Affiliations:** Secretary, Liverpool Council of Voluntary Aid. 2 Exchange Street East, Liverpool


*To the Editor of* The Hospital.

### The Compulsory Removal of Phthisical Patients.

*To the Editor of* The Hospital.

Sir,—In your current issue of The Hospital, on p. 252, you have a note on the compulsory removal of phthisical patients. The paragraph concludes with the statement that "At present public health authorities have no power to enforce isolation or removal . . ." It may interest you to know that under local Acts at least two county boroughs have such powers—namely, Liverpool Corporation and the St. Helens Corporation. There may be others of which I am unaware.—Yours faithfully,

Frederic G. D'Aeth,
Secretary, Liverpool Council of Voluntary Aid.
2 Exchange Street East, Liverpool, June 19, 1916.

